# Supplementary material for: Sleep quality and its correlates among undergraduate medical students in Nepal: A cross-sectional study
Source: PLOS Glob Public Health. 2022 Feb 18;2(2):e0000012. doi: 10.1371/journal.pgph.0000012 (PMC10021869; doi:10.1371/journal.pgph.0000012)
Supplement: Attachment — Submitted filename: Response-to-Reviewer_Sleep_quality_KP and et al..docx [file pgph.0000012.s002.docx]

1^st^ September 2021

Manuscript ID: PGPH-D-21-00378

**Manuscript Title:** Sleep quality and its correlates among undergraduate medical students in Nepal: a cross-sectional study

**Authors:** Kiran Paudel^1,2*^, Tara Ballav Adhikari^2,3,4^, Pratik Khanal^1^, Ramesh Bhatta^5^, Rajan Paudel^1^, Sandesh Bhusal^1,2^, Prem Basel^1^

**Author’s affiliations:** ^1^Institute of Medicine, Tribhuvan University, Maharajgunj, Kathmandu, Nepal**;** ^2^NCD Watch Nepal, Maharajgunj, Kathmandu, Nepal**;** ^3^Department of Public Health, Section for Global Health, Aarhus University, Aarhus, Denmark**;** ^4^Nepal Development Society, Bharatpur-10, Chitwan, Nepal**;** ^5^Asian College for Advanced Studies, Purbanchal University, Satdobato, Lalitpur, Nepal

To

Editorial office

PLOS Global Public Health

**Subject: Response to the editorial and reviewer’s comments for the manuscript reference number:** [PGPH-D-21-00378]

Dear Editor,

Referring to your letter dated August 2, 2021, we thank you for giving us this opportunity to submit our revised research article and responses to reviewers’ comments and suggestions. We thank the editor and reviewers for investing their valuable time in reviewing our paper. We are pleased that there is interest in the topic and are grateful for the constructive comments.

We have addressed the comments point-by-point and have made corresponding changes in the manuscript in track changes. As a result, we feel the changes have improved the manuscript, and we hope it now meets your and the reviewer’s satisfaction. If there are any further comments or clarifications, we will be happy to address them. Below we have responded separately to each point raised. Also we have attached this response letter along with submitted files.

On behalf of all the authors,

Kiran Paudel

[kiranpaudel59@gmail.com](mailto:kiranpaudel59@gmail.com)

**Editor’s comments:**

**In your ethics statement, please state whether participation in the study was voluntary and students were free to opt out at any time. In addition, please state whether the ethics committee approved the use of a digital consent.**

Thank you editor for reminding the ethics statements. We acknowledge the editors comments and added on it on our manuscript as:

“Ethics committee approved the use of digital consent. Study objectives were explained in the Google forms, and e-informed consent was taken from all the participants before the data collection. Study participants were participated voluntary and students were free to opt out at any time.”

*(Page no. 4, Methods, Population)*

**We note that several of your files are duplicated on your submission. Please remove any unnecessary or old files from your revision, and make sure that only those relevant to the current version of the manuscript are included.**

Thank you for the suggestion. Technical problem has been resolved. Now, clean manuscript, manuscript in track changes and reviewer’s report has been uploaded as per editor assigned.

**In the online submission form, you indicated that [Insert text from online submission form here]. All PLOS journals now require all data underlying the findings described in their manuscript to be freely available to other researchers, either 1. In a public repository, 2. Within the manuscript itself, or 3. Uploaded as supplementary information.**

Thank you for highlighting the importance of data set. We have uploaded the data set as supplementary information.

**Reviewers’ reports:**

**Reviewer #1:**

**Comment#1: Authors have mentioned that the students having communication difficulties were excluded. However, the study participants were chosen randomly from college list, how communication difficulties were assessed?**

**Response#1:** Thank you for the suggestion. By communication difficulties we meant study participants who didn't have internet access or who could not be reached either due to lack of interest or they were not checking their emails. We understand that term "*communication difficulties*" might cause confusion. So, we've rephrased that part as:

“Selected participants who didn't respond to our request could not be included in the study. There could be multiple reasons for this, some of which being not having internet access, lacking interest to participate or not checking their emails. Those who did not respond after two times follow up were excluded from the study.” *(Page number: 4, 5 Methods, study population)*

Also, we have mentioned that those not responding were reminded through messaging application wherever possible.

**Comment#2: All the study tools were self-administered through internet, what was the reason to exclude students who had communication difficulty**.

Response**#**2 Participants who we emailed and did not respond in two times follow up were excluded. This is written as “Those who did not respond after two times follow up were excluded from the study.”

(*Page 5; Methods, Study population*)

**Comment#3 Authors have not explained and mentioned how they have addressed the selection bias. Students having higher grades were selected in the study which could lead to the bias as the possibility of studying more hours and sleeping less (poor quality sleep) is higher in students having higher grades**.

Response**#**3 we suppose this comment is related to “selection of study population and students with higher scores were selected for participation each yea" on *sampling* section. The students were not selected according to their higher "grades" as reviewer has mentioned. The roll number of students were listed in a column in Microsoft Excel and in next column, random values were generated using *rand* command. The values (scores) obtained using the rand command were sorted in descending order and those getting the higher random values (scores) were selected in the study and emailed for participation.

We acknowledge the reviewers comments and this is written as: “Using the rand command in Excel, random values were obtained for each students and those with highest value obtained were selected for the participation.”

*(Page 5: Methods; Sampling)*

**Comment#4 More studies could be added to the discussion section of the study.**

Response**#**4 Thank you for the suggestion. We have added more relevant studies to support our discussion.

The literature added to the discussion are as under:

Bawo O. James, Joyce O. Omoaregba OOI. Prevalence and correlates of poor sleep quality among medical students at a Nigerian university. Ann Niger Med. 2011;5(1):1–5.

Rathi, Anurag; Ransing, Ramdas Sarjerao; Mishra, Kshiord Kumar; Narula N. Quality of Sleep among Medical Students: Relationship with Personality Traits. J Clin Diagnostic Res. 2018;12(9):1–4.

*(Page 11; Discussion)*

**Reviewers #2:**

**1. Summary**

The aim of the study was to determine the prevalence of poor sleep quality and its correlates among medical students in a government medical college in Nepal. The authors report a relatively high prevalence of poor sleep quality (38.2%), and identify a number of factors that correlate with this outcome. The reported prevalence is similar or lower than those reported by other studies. The factors influencing sleep quality are largely those that have been previously investigated by other studies, and except for internet addiction and smoking habits, the results align with the literature.

The strengths of the study include their robust methods in relation to the research question, detailed discussion of their results and the conclusions and recommendations being supported by the data presented.

A major weakness of this manuscript is the introduction in two different ways. First, it fails to lay out the reasons or the precise motivation for undertaking this study, since there is recent available literature from Nepal and other countries on sleep quality in medical students, and well as their correlates. In the absence of specific research gaps being highlighted, it is difficult for a reader to evaluate how this manuscript advances our understanding of this area of research. Second, there are several examples of where the reference provided fails to back up the facts stated. I have provided more details below. A third weakness relates to the quality of language used in this manuscript (examples provided below). My overall recommendation is therefore to address these two major weaknesses, especially towards highlighting the advance this paper makes, as well as language editing to clarify important points

Response:

There is limited evidence on the information regarding the sleep quality among the medical students in Nepal. Our study is conducted in a public medical college- the first of its kind while the previous published studies were conducted in private medical colleges. We believe that this study will contribute to the synthesis of existing evidence and highlight the problem of poor sleep quality among this population.

Thank you for flagging the literature from Nepal in the topic of our study. We highly appreciate your effort. We conceptualized our study in 2020 December and implemented our study in March 2021. At that moment, the flagged article was not published. The article was published in July 21, 2021 after the submission of our manuscript. However, following the reviewer’s important remarks we have cited the article in introduction.

**2. Major issues**

Abstract

**Comment#5 In conclusion, the authors only highlight depressive symptoms as an important correlate of poor sleep quality, even though other factors are also described in the results. I would recommend to change this to provide a more balanced conclusion based on what the authors report as their results**

Response**#5:** Thank you for the suggestion we have added this in our abstract section as below:

“Medical students of fourth year, current alcohol users and those who did not have good academic achievement had poor sleep quality. A special attention on these population sub-groups is thus needed for enhancing sleep quality.”

*(Page 2; Abstract)*

**Introduction**

**Comment#6 The following references do not match the facts stated: 5 (not a WHO report), 7, 8, 9 (in paragraph 3)**

Response**#6:** Thank you for pointing this out. We have corrected the reference 5 while 7, 8 and 9 is correctly cited.

*(Page 3; Introduction)*

**The following statements need clarifications:**

**Comment# 7 “Other studies reporting 50 to 70 million people to be chronically suffering from sleep disorders (6)” - clarify that this is only in the American population**

Response**#7:** Thank you for the suggestion and it has been done accordingly.

“Studies suggested that 50 to 70 million American people were chronically suffering from sleep disorders.”

*(Page 3; Introduction)*

**Comment# 8 “Proper sleep aids in the well-functioning of the brain” – reword to “optimum functioning of the brain”**

Response#8 Thank you for the suggestion, we have revised as “optimum functioning of the brain.”

*(Page 3; Introduction)*

**Comment# 9 “improve knowledge and grasp new things” – reword to “grasp new concepts”**

Response#9 Thank you! We have reworded it as “grasp new concepts.”

*(Page 3: Introduction)*

**Comment#10 Clarify** – **“Medical students are prone to developing stressful academics and rigorous medical education”**

Response #10 Thank you for the suggestion. The statement has been clarified as:

“Medical students are prone to stress because of its highly demanding professional roles and academic requirements.”

*(Page 3: Introduction)*

**Comment #11:** Clarify – “Some studies revealed that over 70% of the students face some sort of sleep-related problems” – how does this related to the previous statement which provide ranges between 24-70%? Also note that REF #12 is only a preliminary study, hence the authors must exercise caution while referring to the statistics in the introduction.

Response #11: Thank you for the suggestion. We have now removed the preliminary study from the citation.

*(Page 3; Introduction)*

Comment **#12** Clarify – **“In previous studies conducted in Nepal, the prevalence of poor sleep quality was reported to be 44.2% among medical students and 33.4% among undergraduate non-medical students (14,15) – how was the 33.4% derived since the reference doesn’t list this number?**

Response **#12** Thank you for the suggestion, we have now corrected the prevalence “which is 35.4%.”

*(Page 3; Introduction)*

**Comment #13 Clarify – “long duration of the study year” and “Despite their limited health break”**

Response #13 Thank you for the suggestion. Here, long duration of the study year indicates the duration of the medical (MBBS) course, which is of 5.5 years, higher than any other undergraduate courses (4 years) in Nepal. The limited health break means inadequate vacation and long duration of hospital posting coupled with the academic sessions.

*(Page 4: Introduction)*

Comment**#14 Given the available literature on the topic as referenced by the authors (also see: https://www.ncbi.nlm.nih.gov/pmc/articles/PMC8261762.1/), can the authors clarify the following statement: “there is a paucity of evidence on the sleep quality among medical students in Nepal.”**

Response**#14** Thank you for the suggestion. Our study was conducted and submitted to the journal before the publication of the above mentioned article. Similarly, the published article has limited independent variables like age, sex, and study years only. In our study, we have observed the association of more independent variables such as gender, ethnicity, current smoking and alcohol use, academic performance, depression and smartphone addiction with the outcome variable (sleep quality). Thus, our study has a different research question compared to the published literature in Nepal. Also, the study setting is different to the published literature as our study was conducted in a public medical college. So we expect that this study does have merit with evidence on sleep quality among medical students of Nepal.

*(Page 3; Introduction)*

**Methods**

**Comment#15 Clarify** – **“Participants who had communication difficulty during the study period and below 18 years old.” – how did you define communication difficulty and is this an exclusion criterion? The sentence is incomplete**.

Response#15 Thank you for the suggestion. We have already responded this query above (See response to the Reviewer 1’s comment)

**Comment#16 Clarify** – “**In the excel sheet roll no of the students were kept on one column of each year, and in the next column, a random (rand) command was used, and students with higher scores were selected for participation each year.” – what is meant by higher scores? The method by which the 250 participants were selected from across the 1st-5th year students is not clear.**

Response**#16** Thank you for the suggestion. We have already responded this query above (See response to the Reviewer 1’s comment)

**Comment#17 The study tools used should be described in a little more detail in terms of their use in global and local settings, whether they are validated for the setting in which they have been used, how was validity checked in case not, and how were the cut-offs identified for categorizing into various categories.**

Response#17 The tools we adopted have been used in the similar settings and similar study population in Nepal. For instance, the sleep quality tool was used by Bhandari PM et al. among undergraduate non-medical students in a study published in BMC Psychiatry (<https://bmcpsychiatry.biomedcentral.com/articles/10.1186/s12888-017-1275-5>). Similarly, the same tool was used by Sundas N et al. among undergraduate medical students of a private medical college (<https://www.ncbi.nlm.nih.gov/pmc/articles/PMC7654448/>). Likewise, the tool used to assess smart phone addiction was previously used by Karki S et al. among undergraduate medical students (<https://doi.org/10.1186/s12888-020-02507-1>).

*(Page no; 6 Methods, Study tools)*

We calculated our cutoffs using the categories suggested by the study team who developed the tool. For instance, the cut off of Pittsburg Sleep Quality Index (PSQI) used for measuring sleep quality was suggested by Buysse DJ et al. (<https://pubmed.ncbi.nlm.nih.gov/2748771/> ). The article has been cited by more than 23 thousand literature. Also written in the Methods sections (Study tools) to the respective tool description like “These domains produced a global score, ranging from 0 to 21, where scores greater than five was used to determine the poor sleep quality”.

**Comment#18**

**While analyzing data, why was the outcome categorized and logistic regression the only method chosen? Since categorization is known to reduce the power of the analysis, I would recommend the authors present results from both linear and logistic regressions using continuous and categorical data both, to enable a further refinement in our ability to interpret the associations
- Clarify – “The adjusted odds ratio was calculated at a 95% confidence interval (CI)” – language editing?**

Response**#**18 Thank you for the suggestion. Although we respect the reviewer’s comment, we would like to report the study findings in the manuscript using multivariable logistic regression. This is because, the outcome variable is interpreted based on categories suggested in the original study. Also, we have found sufficient literature which use multivariable logistic regression to identify the correlates of poor sleep quality. Acknowledging the reviewer’s suggestions, we did linear regression and found similar results i.e. depression was a significant correlate while smart phone addiction was not.

Here is a list of similar studies which have reported inferential statistics using binary logistic regression.

Wondie, T., Molla, A., Mulat, H. *et al.* Magnitude and correlates of sleep quality among undergraduate medical students in Ethiopia: cross –sectional study. *Sleep Science Practice* **5,** 7 (2021). Available from: <https://doi.org/10.1186/s41606-021-00058-2>

Lemma, S., Gelaye, B., Berhane, Y. *et al.* Sleep quality and its psychological correlates among university students in Ethiopia: a cross-sectional study. *BMC Psychiatry* **12,** 237 (2012). <https://doi.org/10.1186/1471-244X-12-237>

**Comment#19 Also, a description of the confounding variables used in adjusted analysis is missing, along with a description of why these variables can potentially confound the relationship between exposures and outcomes**

Response**#19** Thank you for the suggestion. Analysis was done by adjusting variables namely depression, smart mobile phone addiction, current alcohol intake status, current smoking status, study year, study choice, academic achievement and self-reported health problem. This has been added as;

“In bi-variable logistic regression analysis; variables namely depression, smart mobile phone addiction, current alcohol intake status, current smoking status, study year, study choice, academic achievement and self-reported health problems had p value less than 0.1. These variables fulfilled minimum requirements for further multivariable logistic regression and were thus fitted in the regression model.”

*(Page no 7; Data analysis)*

**Discussion**

**Comment#20 In places where no references are provided (see example below), the authors should state that it is a speculation**

Response**#20** We have now revised as per you suggestion on the discussion section on second paragraph earlier and now in first paragraph.

- “The possible reasons for the variability across the countries could be due to differences in sampling technique, year of medical school, and exposure to social environment.”

*(Page 12; Discussion)*

**#Comment 21 In cases of such speculation, it would help for the authors to expand on their thought. For the above example, what specific sociocultural factors and differences do the authors have in mind that could lead to differences in sleep quality among medical students?**

**#**Response 21 This is our interpretation for the country wise variation in sleep quality. Therefore we have used the word **‘could be’** as our speculation for possible explanations.

**Comment#22**

**This is applicable to the rest of the discussion as well, whenever references are not provided.
In comparing sleep quality between first- and fourth-year students, the authors make a statement of higher academic and clinical load on the fourth-year students. What happens to 5th year students? What are the protective factors for the 5th year students that bring down the odds from 3.6 in 4th year to 1.8 in 5th year students?**

Response**#**22 Thank you for the queries and we explained as “According to the curriculum, students in the fourth year have to face series of examinations which puts the students in a tough mental pressure. Additionally, failure in the fourth year leads to discontinuation of hospital based internship. However, students in the fifth year, need not face examinations **which might have** contributed to such differing results.”

*(Page 11; Discussion)*

**Comment#23What are the authors thoughts on why smartphone addiction was not associated with sleep quality in this sample, as against what the literature states as the authors rightly point out?**

Response**#**23 Thank you for the suggestion. Existing literature however shows a mix of results on the association between smartphone addiction and sleep quality. The explanation to this finding thus needs further study in a larger sample population.

On behalf of the research team, I would like to thank the reviewers in improving the quality of our manuscript and we believe that the manuscript if accepted will be of wide attention to the readers in Nepal and similar settings.

Regards,

Kiran Paudel

[kiranpaudel59@gmail.com](mailto:Kiranpaudel59@gmail.com)
