# Supplementary material for: Sleep quality and its correlates among undergraduate medical students in Nepal: A cross-sectional study
Source: PLOS Glob Public Health. 2022 Feb 18;2(2):e0000012. doi: 10.1371/journal.pgph.0000012 (PMC10021869; doi:10.1371/journal.pgph.0000012)
Supplement: Attachment — Submitted filename: Response_to_editor.docx [file pgph.0000012.s003.docx]

3^rd^ Nov 2021

**Manuscript ID: PGPH-D-21-00378**

**Manuscript Title:** Sleep quality and its correlates among undergraduate medical students in Nepal: a cross-sectional study

**Authors:** Kiran Paudel^1,2*^, Tara Ballav Adhikari^2,3,4^, Pratik Khanal^1^, Ramesh Bhatta^5^, Rajan Paudel^1^, Sandesh Bhusal^1,2^, Prem Basel^1^

**Author’s affiliations:** ^1^Institute of Medicine, Tribhuvan University, Maharajgunj, Kathmandu, Nepal; ^2^NCD Watch Nepal, Maharajgunj, Kathmandu, Nepal; ^3^Department of Public Health, Section for Global Health, Aarhus University, Aarhus, Denmark; ^4^Nepal Development Society, Bharatpur-10, Chitwan, Nepal; ^5^Asian College for Advanced Studies, Purbanchal University, Satdobato, Lalitpur, Nepal

To

Editorial office

PLOS Global Public Health

**Subject: Response to the editorial and reviewer’s comments for the manuscript reference number: [PGPH-D-21-00378]**

Dear Editor,

Referring to your letter dated October 26, 2021, we thank you for giving us this opportunity to submit our revised research article. We thank the editor for investing their valuable time in reviewing our paper. We are pleased that there is interest in the topic and are grateful for the constructive comments.

We have addressed the comments point-by-point and have made corresponding changes in the manuscript in track changes. As a result, we feel the changes have improved the manuscript, and we hope it now meets yours’s satisfaction. If there are any further comments or clarifications, we will be happy to address them. Below we have responded separately to each point raised. Also we have attached this response letter along with submitted files.

On behalf of all the authors**,**

Kiran Paudel

[kiranpaudel59@gmail.com](mailto:kiranpaudel59@gmail.com)

**Editor’s comments:**

**We have noticed that you have uploaded supporting information but you have not included a list of legends.  Please add a full list of legends for all supporting information files (including figures, table and data files) after the references list.  We have noticed that you have uploaded raw data files as supporting information but you have not included them in the list of legends.  Please add a citation of this SI data files into the list of legends of your supporting information files.**

We have added the full list of legends for all supporting information files (data file) after the reference list as.

**“Supporting Information**

***S1 Dataset****. Data underlying the results of the study.*

[*Sleep_Quality_Dataset_Kiran_and_et_al.xlsx*](https://www.editorialmanager.com/pgph/download.aspx?id=18350&guid=08955aa9-0682-46b1-a1e0-ec0f64c01c9e&scheme=1)

*Data file in xlsx*. “

**Add the possible selection bias that may have occurred due to non-response in the limitations.**

Thank you editor for suggesting us to write there might be possible selection bias that may have occurred due to non-response in the limitation. We acknowledge the editor’s comments and added on it on our manuscript as:

“*There might be selection bias that may have occurred due to non-response*”.

(*Page number: 13*)

**While you have responded that the tools have been used in Nepal in the past, it is unclear whether tools were validated in the language and population. Please add details regarding validation.**

Thank you editor for flagging this issue whether the tools were validated in the language and population or not. Tools we used were in English language and were validated in the language and same population like ours. During the pretesting no one reported the difficulties in understanding the questionnaire and decided to administer questionnaire in English version. Being the study population of university level and also the subjects taught in English, it has been easy for the participants to understand,

**Describe how each of the confounding factors information was collected in the methods section.**

We have described on methods section; how the confounding factors information was collected as:

“In bi-variable logistic analysis, variables namely depression, smart mobile phone addiction, current alcohol intake status, current smoking status, study year, study choice, academic achievement, and self-reported health problems were found to have p-value less than 0.1. These variables fulfilled minimum requirements for further multivariable logistic regression and was thus fitted in the final regression model. Multivariable logistic regression analysis was conducted to determine the statistically significant association between explanatory variables and outcome variables. The adjusted odds ratio was calculated at a 95% confidence interval (CI), and a p-value less than 0.05 was considered statistically significant.”
