# Supplementary material for: Sleep quality and its correlates among undergraduate medical students in Nepal: A cross-sectional study
Source: PLOS Glob Public Health. 2022 Feb 18;2(2):e0000012. doi: 10.1371/journal.pgph.0000012 (PMC10021869; doi:10.1371/journal.pgph.0000012)
Supplement: Attachment — Submitted filename: Response_to_editor.docx [file pgph.0000012.s004.docx]

**Date:** 14^th^ November 2021

**Manuscript ID:** PGPH-D-21-00378

**Manuscript Title:** Sleep quality and its correlates among undergraduate medical students in Nepal: a cross-sectional study

**Authors:** Kiran Paudel^1,2*^, Tara Ballav Adhikari^2,3,4^, Pratik Khanal^1^, Ramesh Bhatta^5^, Rajan Paudel^1^, Sandesh Bhusal^1,2^, Prem Basel^1^

**Author’s affiliations:** ^1^Institute of Medicine, Tribhuvan University, Maharajgunj, Kathmandu, Nepal; ^2^NCD Watch Nepal, Maharajgunj, Kathmandu, Nepal; ^3^Department of Public Health, Section for Global Health, Aarhus University, Aarhus, Denmark; ^4^Nepal Development Society, Bharatpur-10, Chitwan, Nepal; ^5^Asian College for Advanced Studies, Purbanchal University, Satdobato, Lalitpur, Nepal

To

Editorial office

PLOS Global Public Health

**Subject:** Response to the editorial and reviewer’s comments for the manuscript reference number: PGPH-D-21-00378

Dear Editor,

Referring to your letter dated November 9, 2021, we thank you for giving us this opportunity to submit our revised research article. We thank the editor for investing her valuable time in reviewing our paper. We are pleased for your constructive comments.

We have addressed the comments point-by-point and have made corresponding changes in the manuscript in track changes. As a result, we feel the changes have improved the manuscript, and we hope it now meets your satisfaction. Below we have responded separately to each point raised. Also we have attached this response letter along with submitted files.

On behalf of all the authors**,**

Kiran Paudel

[kiranpaudel59@gmail.com](mailto:kiranpaudel59@gmail.com)

**Editor’s comments:**

**Comment1: Please explain how the possible selection bias would have affected the study results. That is, if the non-responders are likely to have better or worse sleep quality than responders, how does that affect the study results.**

**Response1: Thank you for the suggestion, we have explained as follows:**

**“**There might be respondent bias as the findings were self-reported and based on subjective scale, resulting in underestimating, and overestimating their behaviors. There might be selection bias that may have occurred due to non-response. **Due to selective non-response bias, the prevalence of self-reported variables such as smartphone addiction, alcohol consumption, and tobacco consumption may be significantly underestimated. Students who use tobacco and alcohol may be among those who were not chosen and who did not respond.”**

***Limitation page no 13; Second Paragraph***

**Comment2: In the methods (not in the analysis part), explain how the variables (depression, smart phone addiction, current alcohol intake status, current smoking status, study year, study choice, academic achievement, and self-reported health problems) were collected. That is, explain how the depression, smartphone addiction or current alcohol use were ascertained or defined in the study.**

**Response2: Thank you Editor for your valuable suggestions. Now we have elaborated this in method section abiding your feedback. The variables like depression, smartphone addiction, current alcohol intake status, current smoking status, study year, study choice, academic achievements, and self-reported health problems were collected as follows:**

A self-administered questionnaire was distributed to the participants through their email addresses. A reminder was sent through Viber, WhatsApp, and Facebook; wherever possible in case of non-response to the email. The socio-demographic section of study tools consisted of information about participants’ sex (male, female), ethnicity (Brahmin/Chettri, Madhesi, Adiwasi/Janjati, Dalit), current living residence (in hostel, with family, alone), year of the study (first, second, third, fourth, final), family income (less than 20,000, 20,000-50,000, 50,000 and above), academic achievement (pass, fail), and study choice (own preference, family pressure). To assess the academic performance of the students, the outcome of the board examination in the previous year was recorded, those who have succeeded were grouped as passed and others as failed. For the first-year students, the outcome of the previous internal examination was recorded. Substance-related factors (current smokers and current alcohol users) were assessed by WHO ASSIST (Alcohol Smoking and Substance Involvement Screening Tool [21]. We grouped students based on their use of tobacco and alcoholic beverages in the past 30 days. Current tobacco users are defined as the students who had consumed a tobacco product either smoking or chewing at least once. Similarly, students who had consumed any alcoholic beverages at least once in the past 30 days were defined as current alcohol users.

For the assessment of depression, a nine-item Patient Health Questionnaire-9 (PHQ-9) was used. The tool recorded the frequency of symptoms of depression during the past two weeks. Responses were recorded as “not at all”, “several days”, “more than half the days” or “nearly every day” and were scored between 0-3. A composite score of 0-27 was generated, and a score ≥ 10 was considered as a depressive disorder [22].

For the assessment of smartphone addiction, ten items Smartphone Addiction Scale Short Version (SAS-SV) was used. On self-reporting six-point Likert scale (1: “strongly disagree”, 2: “disagree”, 3: “weakly disagree”, 4: “weakly agree”, 5: “agree”, and 6: “strongly agree”). The scores were summed, and score higher than 32 was considered as problematic smartphone addiction [23].

Sleep quality was assessed by using Pittsburgh Sleep Quality Index (PSQI). It differentiated the sleep quality over the past month based on seven domains: subjective sleep quality, sleep latency, sleep duration, habitual sleep efficiency, sleep disturbances, use of sleep medication, and daytime dysfunction. PSQI consists of 19 items and produced a score of 0 (no difficulty) to 3 (severe difficulty) on these seven domains. These domains produced a global score, ranging from 0 to 21, where score greater than five was used to determine the poor sleep quality [24].

Inclusion of the individuals independent variables that could affect sleep quality was done by referring to the previous studies [14–16].

**References**

14.	Pilcher JJ, Walters AS. How sleep deprivation affects psychological variables related to college students’ cognitive performance. Journal of the American College Health Association. 1997;46: 121–126. doi:10.1080/07448489709595597

15.	Bawo O. James, Joyce O. Omoaregba OOI. Prevalence and correlates of poor sleep quality among medical students at a Nigerian university. Annals of Nigerian Medicine. 2011;5: 1–5. doi:10.4103/0331-3131.84218

16.	Almojali AI, Almalki SA, Alothman AS, Masuadi EM, Alaqeel MK. The prevalence and association of stress with sleep quality among medical students. Journal of Epidemiology and Global Health. 2017;7: 169–174. doi:10.1016/j.jegh.2017.04.005

21.	Heslop K, Ross C, Osmond B, Wynaden D. The Alcohol Smoking and Substance Involvement Screening Test (ASSIST) in an Acute Mental Health Setting. International Journal of Mental Health and Addiction. 2013;11: 583–600. doi:10.1007/s11469-013-9428-3

22.	Manea L, Gilbody S, McMillan D. A diagnostic meta-analysis of the Patient Health Questionnaire-9 (PHQ-9) algorithm scoring method as a screen for depression. General Hospital Psychiatry. 2015;37: 67–75. doi:10.1016/j.genhosppsych.2014.09.009

23.	Kwon M, Kim DJ, Cho H, Yang S. The smartphone addiction scale: Development and validation of a short version for adolescents. PLoS ONE. 2013;8: 1–7. doi:10.1371/journal.pone.0083558

24.	Buysse DJ, Reynolds CF, Monk TH, Berman SR KDJ. The Pittsburgh Sleep Quality Index: a new instrument for psychiatric practice and research. Psychiatry Research. 1989;28: 193–213.

*Methods; Page no 5, 6*

**Comment3: Check the language, grammar and typos throughout the manuscript.**

**Respone3:** We have checked the language, grammar, and typos throughout the manuscript with the help of an English language experts.

**Comment4: Add reference to the sentence (page 3, second para)**

Response4: Thank you for the suggestion. We have added the following references to the page 3, second para.

**References**

11.	Webb E, Ashton C, Kelly P, Kamali F. Alcohol and drug use in UK university students. The Lancet. 1996;348: 922–925. doi:10.1016/S0140-6736(96)03410-1

12.	Ban DJ, Lee TJ. Sleep Duration, Subjective Sleep Disturbances and Associated Factors Among University Students in Korea. J Korean Med Sci. 2009;16: 475–480. doi:10.3346/jkms.2001.16.4.475

13.	Tsai L-L, Li S-P. Sleep patterns in college students: Gender and grade differences. Journal of Psychosomatic Research. 2004;56: 231–237. doi:10.1016/S0022-3999(03)00507-5

**Comment5: A higher prevalence of poor sleep quality among medical students than non-medical students and general population has also been reported.**

**Response5:** Thank you for the suggestion. Also, we have added a reference given below to this sentence.

**References**

5.Azad MC, Fraser K, Rumana N, Abdullah AF, Shahana N, Hanly PJ, et al. Sleep disturbances among medical students: A global perspective. Journal of Clinical Sleep Medicine. 2015;11: 69–74. doi:10.5664/jcsm.4370

**Comment6: Incomplete sentence. Rephrase. (Page 4, para 1)**

**Despite their limited health break due to inadequate vacation and long duration of hospital posting coupled with academic sessions and stressful lifestyle.**

**Response6:** Thank you for the suggestion, we have rephrased as:

“Despite inadequate vacation and long duration of hospital posting along with academic sessions and stressful lifestyle, there is limited evidence regarding the burden of poor sleep quality among medical students in Nepal.”

***Introduction; Page no 4***

**Comment7: Please edit the sentence as below. Also explain the why the students at public funded medical college will be different from the private funded college. (Page 4, para 1)**

**Our study in a public-funded medical college is first of its kind as the previous published studies were conducted in private medical colleges.**

**Response7:** Now, we have rephrased it as “Our study in a public funded medical college is first of its kind as the previous published studies were conducted in private medical colleges.”

And also explain as:

“Students from various parts of the country, with varying socioeconomic status, attend a publicly funded medical college, creating diversity among them that would be interesting to study. Students with the highest academic standing are considered to study at a publicly funded medical college. Due to the high flow of patients, students have to perform more clinical duties.”

***Introduction; Page 4***
